# Isothermal Microcalorimetry to Investigate Non Specific Interactions in Biophysical Chemistry

**DOI:** 10.3390/ijms10083283

**Published:** 2009-07-28

**Authors:** Vincent Ball, Clarisse Maechling

**Affiliations:** 1 Institut National de la Santé et de la Recherche Médicale, Unité mixte de recherche 977, 11 rue Humann, 67085 Strasbourg Cédex, France; 2 Université de Strasbourg, Faculté de Chirurgie Dentaire, 1 Place de l’Hôpital, 67000 Strasbourg, France; 3 Laboratoire d’Innovation Thérapeutique, Unité Mixte de Recherche 7200 CNRS - Université de Strasbourg, Faculté de Pharmacie, 74 route du Rhin BP 60024, F-67401 ILLKIRCH Cedex, France; E-Mail: clarisse.maechling@unistra.fr (C.M.)

**Keywords:** non-specific interactions, isothermal titration calorimetry

## Abstract

Isothermal titration microcalorimetry (ITC) is mostly used to investigate the thermodynamics of “*specific” host-guest interactions* in biology as well as in supramolecular chemistry. The aim of this review is to demonstrate that ITC can also provide useful information about non-specific interactions, like electrostatic or hydrophobic interactions. More attention will be given in the use of ITC to investigate polyelectrolyte-polyelectrolyte (in particular DNA-polycation), polyelectrolyte-protein as well as protein-lipid interactions. We will emphasize that in most cases these “non specific” interactions, as their definition will indicate, are favoured or even driven by an increase in the entropy of the system. The origin of this entropy increase will be discussed for some particular systems. We will also show that in many cases entropy-enthalpy compensation phenomena occur.

## 1. Introduction: Specific Versus Non-specific Interactions, What Is the Best Criterion?

One of the fundamental challenge in biophysical chemistry, as well as in all physicochemical events implying a solvent, is to attribute the contribution of different non-covalent interactions (electrostatic in the most general sense, solvation and hydrophocic interactions, hydrogen bonding, Van der Waals interactions) to the free energy change of a given molecule upon its interaction with a binding partner. This is also of prime interest in pharmacology, where the design of drugs aimed at binding to a given target relies on engineering contributions that will increase the absolute enthalpy change upon binding as well as contributions that will increase the entropy change [1]. Another challenge is to investigate how conformational changes in the tridimensional structure of a protein, DNA or RNA can modify the interaction between this molecule and its binding partner [2]. This last point is directly related to the problem of cooperativity [3] which also emerges in supramolecular chemistry. This is not surprising because both biological molecules and synthetic supramolecules interact via the same interplay of “non-covalent” interactions [4]. Finally, the most important challenge is to relate structure (the high resolution X-ray or NMR structure of more and more proteins and nucleotides are available) to the “binding affinity” of these molecules with their binding partners. This means that the “binding affinities” have to be accurately measured for as many as possible systems to get a clear picture of structure-energy relationships, which is particularly important for the design of new drugs or the design of gene vectors.

In this review, on purpose, we wish to not use the general terms of “host” and “guest” because these definitions point immediately to a key and lock interaction mechanism and hence to “specificity” (we will recall the definition of this concept later). In the next lines we will discuss a definition of “specific” and “non-specific” interactions, with the aim of reviewing the major contributions of microcalorimetry (in particular isothermal titration microcalorimetry, ITC) in the investigation of the thermodynamics of “non-specific” interactions.

Let us recall that when a transformation takes place in conditions of constant temperature and pressure, the chemical potential of use is the free energy. Its change between the initial and the final state is given by:

(1) $$\Delta G^{{}^{\circ}}=\Delta H{}^{\circ}-T.\Delta S{}^{\circ}$$

where Δ*H*° and Δ*S*° are the change in the enthalpy and entropy of the system under standard conditions (symbolised by the superscript °). The standard conditions refer just to the fact that the experiment is performed at a “standard” pressure of 1 bar and that the activity of each constituent is equal to 1. The same equation (1) is valid in non standard conditions. Equation (1) shows that a given “binding affinity” and hence a given value of Δ*G* can be obtained with different contributions of enthalpy and entropy changes. The affinity is indeed the change in free energy with its sign changed. Hence a high affinity means an important decrease in the free energy upon binding. Anyway, the condition for a spontaneous evolution of a system is that: Δ*G*^0^ < 0. It has to be noted that most of the metabolic transformations proceed in conditions of non spontaneity ( $\Delta G_{1}^{{}^{\circ}}>0$) because they are coupled with at least one transformation which occurs spontaneously [5] ( $\Delta G_{2}^{{}^{\circ}}<0$) with the constraint that:

(2) $$\Delta G_{1}^{{}^{\circ}}+\Delta G_{2}^{{}^{\circ}}<0$$

The free energy change upon binding is experimentally accessible (provided the interaction is an equilibrium process!) via the measurement of the macroscopic equilibrium constant *K*, according to the well known relationship:

(3) $$\Delta G^{0}=-R.T.\text{ln}K$$

where *R* is the gas constant (8.314 J.mol^−1^.K^−1^) and *T* the absolute temperature in Kelvin.

This means that every experimental method able to determine the binding constant of the investigated transformation will give access to the free energy change associated with this transformation. Basically if the transformation follows the chemical equation:

(4) A+B⇔C

the global free energy change will be given by the difference in the chemical potential (the molar Gibbs free energy) between the product (C) and the reactants (A and B):

(5) $$\Delta G=\mu_{C}-\mu_{A}-\mu_{B}$$

When the energy changes are only of chemical nature, i.e., in the absence of applied external fields, using the relationship between the chemical potential and the activity, equation (5) becomes:

(6) $$\Delta G=\mu_{C}^{{}^{\circ}}-\mu_{A}^{{}^{\circ}}-\mu_{B}^{{}^{\circ}}+R.T.\text{ln}\left(\frac{a_{c}}{a_{A}.a_{B}}\right)$$

The difference in the standard chemical potential (defined at 1 bar and at an activity of 1), may be written as:

(7) $$\Delta G{}^{\circ}=\mu_{C}^{{}^{\circ}}-\mu_{A}^{{}^{\circ}}-\mu_{B}^{{}^{\circ}}$$

which finally gives:

(8) $$\Delta G=\Delta G{}^{\circ}+R.T.\text{ln}\left(\frac{a_{c}}{a_{A}.a_{B}}\right)$$

The equilibrium conditions correspond to Δ*G* = 0, which gives equation (3). The equilibrium constant *K* of equilibrium (4) being then defined as:

(9) $$K=\frac{a_{C}}{a_{A}.a_{B}}$$

We spent some time to derive such a trivial equation as (9) to show that the equilibrium constant is defined from the *activities* of the species taking part to the equilibrium and not from the concentrations. For solutes of low molecular mass, this is not of major concern because the activity becomes equal to the concentration at infinite dilution (hence practically for small concentrations, typically in the millimolar range). But this does not necessarily hold true any more in the case of macromolecules, like proteins or DNA, because the volume fraction of these macromolecules can be high even at low molar concentration.

The distinction between “specific” and “non-specific” interactions explains the somewhat provocative title of this article. As we will see, it is difficult to put a clear frontier between what is called “specific” and “non-specific”, even if the term “specific” would refer to an interaction that is unique to a couple of binding partners, i.e. to the selectivity of the binding process. In most cases, as we will see, the situation is not as simple. The distinction between these two words is as difficult as the distinction between “interactions of physical” and “interactions of chemical nature”. Similarly, in surface science it is difficult to distinguish between specific and non-specific adsorption [6]. Indeed when one considers the interactions between amino acids and surfaces, the adsorption can have a specific counterpart even when the adsorption is performed in the gas phase [7]. The contribution of specific interactions has been demonstrated for the adsorption of glycine on titania [8]. There are many examples in which the specific adsorption of surface potential determining ions modify the surface potential of surfaces, the most well known example being that of silver iodide in presence of surface potential determining ions like I^−^ [9]. The accumulation of counterions (i.e. of opposite sign to that of the permanent charges at the interfaces) in the electrical diffuse double layer may then be considered as “non-specific”. The condensation of counterions around strongly charged polyelectrolytes may at first glance be considered as non-specific because it originates from the strong electrostatic potential of the polyelectrolyte [10]. By condensation we mean that the counter ions are bound within a few tenths of a nanometer from the polyelectrolyte backbone. The occurrence of ion condensation on polyelectrolytes or on surfaces is the result of a competition between attractive electrostatic forces and the disrupting effect of thermal motion. The strong potential on certain polyelectrolytes originates from the chemical nature of the polyelectrolyte and can hence be considered as a “specific” effect that is due to the nature and the density of the charged groups carried by the polyelectrolyte.

In addition, the nature of the electrolyte can afford some specificity [11], the so called Hofmeister effect. These effects cannot be described simply by considering the electrolyte solution as a continuous medium of dielectric constant ε where the screening of the electrostatic forces is just described by a macroscopic adjustable parameter, the ionic strength of the solution.

**Figure 1.**
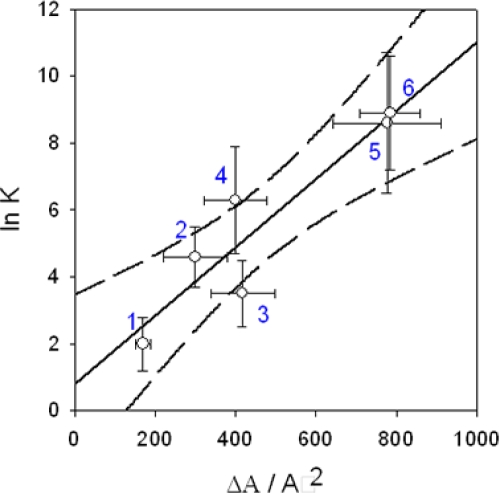
Relationship between the logarithm of the binding constant and the reduction in surface area accessible to the solvent upon binding between two partner molecules. This plot is based on the data in Table 7 of Houk *et al*. [12]. interactions between α-cyclodextrins and organic guests.interactions between catalytic antibodies and organic substrates.interactions between albumins and organic guests.interactions between catalytic antibodies and inhibitors.interactions between enzymes and inhibitors.interactions between antibodies and proteins.The straight line is a linear regression to the data (*r^2^*=0.86) whereas the dashed line corresponds to the limit of the 95 % confidence interval.

When considering interactions in supramoleular chemistry (implying cyclodextrins, or a plethora of synthetic hosts) as well as in biology (antigen-antibody complexes, protein-ligand binding and enzyme substrates complexes), which are all considered as highly “specific” interactions, it has been found out that the free energy change associated with the binding process is proportional to the solvent accessible surface area that is buried upon binding, Figure 1. These investigations have been performed by means of molecular docking [12] and analysis of crystal structures [13].

Despite the great diversity of binding partners, the correlation between *lnK* (and hence the change in free energy) and the area buried from the solvent upon binding is pretty good (*r^2^*=0.86). Does that mean that the most important part of the interaction is related to the area that can be buried upon binding, and hence to the size of the “binding sites”? Or does it mean that the probabilty to have a contribution to the free energy decrease, be it “specific” or “non-specific” is proportional to the area of the part of the molecule that is implied in the molecular recognition process?

All the above examples show that the distinction between “specific” and “non-specific” effects are never clear cut. One can define an upper limit in the binding energy to distinguish “specific” from “non-specific”: this would imply that specificity is equivalent to “high affinity”. For free energy changes greater than a critical value one can define the interaction to have a “specific character” whereas for changes in free energy lower than this threshold, one assumes the interaction to be “non-specific”. It is exactly on this basis that one traditionally separates chemisorption from physical adsorption and more generally “covalent bonds” from secondary interactions. Here again this clear separation may be arbitrary, because there are covalent bonds (established through the fact that the bond can be described by a molecular orbital constructed from atomic orbitals belonging to atoms of the molecule) implying a binding energy (the energy variation between isolated and bond atoms in the gas phase) that is very close to the above mentioned threshold. For instance the binding energy of Li_2_ amounts only to 60 kJ.mol^−1^ [14]. However, this compound is not an aggregate of two Li atoms: The motions of both Li atoms are strongly correlated inside the molecule.

When considering adsorption phenomena, it is clear that the contribution of many weak interactions (each one being of the order of *kT*, ie the average energy due to random collisions with solvent molecules) can lead to an irreversible adsorption, i.e. to high affinity, as in the case of chemisorption. Such assumptions, which become validated by experimental observations [15] and simulations [16] allow one to explain the irreversible character of protein or polymer adsorption at solid–liquid interfaces. This example clearly shows that high affinity can be reached without “specificity”. The previous example of Li_2_ shows that “specificity” can be reached even in the absence of high affinity. Clearly, the criterion of affinity is not satisfactory to separate “specific” from “non-specific” interactions neither is the concept of a given stoichiometry. Many so called “non-specific” binding processes have nevertheless a defined stoichiometry. Indeed adsorption phenomena or ion binding events at surfaces often reach a saturation, but in the common sense most of them are “non-specific” (proteins stick everywhere, counterions can interact with every surface of opposite sign). At saturation of an adsorption process, there is clearly a maximal amount of bound molecules or ions (the sorbent) for a given number of surface sites (indeed it is often very difficult to have access to the density of surface sites particularly if the sorbate is heterogeneous, the situation becomes ideal only in the case of very clean monocristalline terraces) and hence a given stoichiometry. The fact that in some adsorption experiments one not reaches the saturation value does not mean that there is no stoichiometry, it can simply mean that the sorbate is not provided in sufficient amount to cover all the adsorption sites. It has to be noted also that the notion of a 1 to 1 interaction between a sorbate and a site on the surface is a relative notion which depends on the size and the shape of the sorbate. This consideration is at the origin of improvements of the original Langmuir adsorption isotherm in which the surface exclusion function is simply considered to be proportional to the number of adsorbed molecules and hence neglects interactions between triplets and other kinds of multiplets (in a 2D crystal each adsorbed molecule can have up to 6 closest neighbours) [17].

**Figure 2.**
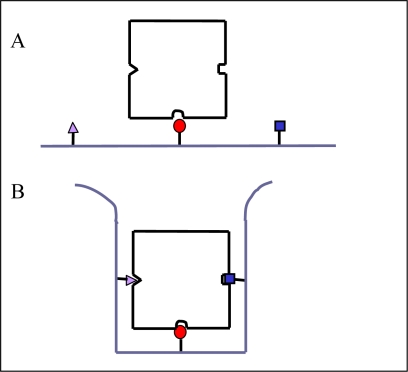
Schematic representation of the difference in geometric disposition between three chemical groups able to interact with complementary receptor sites on a binding partner molecule allowing for only “non-specific” interactions (A) and “specific” interactions (B). Nevertheless “non-specific” interactions can also contribute to increase the binding affinity in the case of B because of desolvation of both molecules upon their contact. In the case of a given biomolecule one can go from situation B to A by a single conformational change.

If neither the criterion of a maximal change in free energy, nor the absence of a saturation in binding can be used to distinguish between “specific” and “non-specific” binding, what can be a reasonable criterion? Let us come back to E. Fisher’s “key and lock” analogy which is the foundation of the idea of specificity. In a “chemical lock” it is neither the geometry alone nor the presence of some given chemical functions which allows this lock to recognize some molecules with some preference with respect to other ones, *it is indeed a combination of both.* The “good” chemical groups have to be present in a given well defined but nevertheless flexible topology. Hence one can say that “specificity” corresponds to a pattern of molecular recognition (Figure 2).

There is an other important point: *Most often the affinity of “non-specific” processes is mainly due to an increase in entropy, whatever its origin* [1]. Hence many processes implying hydrophobic interactions are mainly “non-specific” in nature. Let us recall that the hydrophobic interaction between two aliphatic chains is not due to some preferential interactions between these chains nor to a repulsion between each of them and water, *it is due mainly to an entropy increase of the water molecules upon desolvation of the chains.* This desolvation of course costs some enthalpy.

The difference in the chemical potential of an aliphatic chain with *n* carbon atoms between an apolar solvent and water is proportional to *n* at least for chains having less than 22 carbon atoms [18]:

(10) $$\mu_{Apolar}^{0}-\mu_{water}^{0}=\alpha -\beta .n$$

where α and β are two positive constants. α takes into account for the unfavorable effect the head group of amphiphiles has on its solubility in apolar solvents.

The fact that the reduction in free energy upon transfer from water to an apolar solvent is simply proportional to the chain length is clearly a “non specific” effect: the longer the aliphatic chain the less it is soluble in water.

With this in mind it is now clear that on the surface of:

- a lipid vesicle (with a known chemical composition) even in the presence of phase boundaries (the so called “rafts”),
- on the surface of a mineral oxide,
- along a polyelectrolyte chain,
- along a single stranded oligonucleotide,

there are clearly no such defined recognition patterns. On the other hand, prosthetic groups of proteins like the well know protoporphyrin IX of hemoglobin are “specific” recognition motifs. The amino acids inside the channel of a membrane protein play also the role of a specific recognition motif.

The energetics of this kind of “specific” interactions has been reviewed in excellent fashion by many authors [19–21]. In this article we aim to review how microcalorimetry helps to determine the energetics of binding process implying non specific interactions. Such interactions are “ill defined” in the sense that there is not well defined localization and topology of the interacting chemical groups. In each investigated case, we will try to provide some relevant examples from the older as well as from very recent literature, but without the aim to be exhaustive. We will focus on interactions in which biomolecules are exposed to interfaces. Indeed in this situation, very common in biotechnology, biomolecules having specific binding groups are exposed to chemical groups that do not display a configuration allowing for specific interactions to take place.

In general, there are plenty of experimental methods able to determine the binding constant between two interacting molecules:

- NMR,
- other spectroscopic methods like fluorescence spectroscopy,
- sedimentation velocity and equilibrium,
- equilibrium dialysis [22] (which can be used only when the two binding partners have very different hydrodynamic radii and when one of them is able to diffuse across the pores of the dialysis membrane),
- chromatography,
- capillary electrophoresis
- even electrospray mass spectrometry, ESI [23] (ESI measures the mass increase of the guest molecule upon binding in the dry state, which means that all the contribution of hydrophobic interactions are not taken into account)
- and of course, titration calorimetry [24].

Surface plasmon resonance can be used when one of the binding partners can be immobilized at a solid liquid-interface in contact with a noble metal. The collective modes of motions of the electrons (the surface plasmons) can be exploited to investigate the increase in refractive index which is the consequence of the binding process during which an accumulation of binding molecules occur at the interface [25].

In the next paragraph we will emphasize why calorimetry is really necessary to unravel the enthalpic and entropic contributions of binding processes. Finally, in the last and most important chapter we will highlight how ITC can help to investigate “non-specific” interactions (in the sense defined before) in the fields where molecules relevant to biology are exposed to an interface. Namely we will describe the use of ITC (and sometimes of differential scanning calorimetry, DSC) in the following subfields:

- self assembly of amphiphiles,
- interactions between peptides-drugs with lipid membranes,
- proteins with surfaces,
- peptides with DNA as well as proteins or DNA with polyelectrolytes.

In all these cases the global enthalpy and entropy change which contribute to Δ*G*° [equation (1)] are the result of many subcontributions. The most challenging point, as already mentioned is to unravel each of these contributions. We will conclude by emphasizing the need to investigate very simple model systems to go further in this direction.

## 2. Some Other Fundamental Relationships and the Need to Measure the Enthalpy Change Independently from the Binding Constant

Assuming that the standard enthalpy and entropy changes are temperature independent, one easily derives the van’t Hoff relationship from equations (1) and (3):

(11) $$\frac{\partial \text{ln}K}{\partial T}=\frac{\Delta H{}^{\circ}}{RT^{2}}$$

which shows, that the equilibrium constant increases upon a temperature increase for an endothermic process (Δ*H*° > 0).

Upon the derivation of (11), *the assumption of constant standard enthalpy and constant standard entropy is of paramount importance* and has often been neglected in the literature [26]. It is nevertheless well known that most of the “non-specific” (as well as the “specific”) interactions are occurring with Δ*H*° and Δ*S*° being temperature dependent. This is also true for specific interactions as exemplified by the complexation of Ba^2+^ by 18-crown-6 ether (water being the solvent) [27].

When Δ*H*° is temperature dependant, the investigated transformation implies a change in the heat capacity of the whole system:

(12) $$\Delta C_{P}={\left(\frac{\partial \Delta H{}^{\circ}}{\partial T}\right)}_{P}$$

where the subscript *P* means that the transformation is investigated at constant pressure.

From equation (12) it is clear that after having measured the temperature dependence of C_p_, one can also get the standard enthalpy change associated with the investigated process by means of integration:

(13) $$\Delta H{}^{\circ}=\int_{T_{i}}^{T_{f}}\Delta C_{P}(T).dT$$

where *T_i_* and *T_f_* are the initial and final temperatures respectively.

Hence, DSC allows to measure the enthalpy of protein denaturation which provides an unestimable information about its conformational stability [28,29]. Indeed, upon denaturation, hydrophobic residues hidden from the solvent in the folded state become exposed to the solvent with an increase of the heat capacity [30].

Relying on the validity of the van’Hoff relationship, it appears that measuring the equilibrium constant of a given equilibrium at different temperatures will allow to calculate Δ*H*°. Before the availability of highly sensitive microcalorimeters, many investigators used the temperature dependency of the equilibrium constants to deduce the enthalpy change associated with the binding process. The use of equations (1) and (3) then allow to calculate the corresponding entropy change Δ*S*°. Very sensitive and fast responsive microcalorimeters now allow to alleviate this bias [26].

The knowledge of both Δ*H*° and Δ*S*° is of paramount importance to unravel the different contributions of any interaction process taking part in presence of a solvent (water). The desolvation of hydrophobic surfaces, the hearth of the hydrophobic effect [18,31,32], is a process that increases the number of accessible states of the water molecules and hence an increase in the whole entropy of the system. When one or both of the binding partners lose some of their degrees of motional freedom (vibrational and/or rotational), the entropy of the system will decrease. Hence the global knowledge of the value of the entropy (enthalpy) change does by itself not give the value of its individual contributions [33]. Techniques complementary to calorimetry are thus necessary to decompose the enthalpy and entropy changes into their constitutive parts. This is indeed very challenging as we will see in the particular case of “non specific” interactions. For instance, NMR relaxation experiments allow to compare the mobility of a protein binding site before and after the binding event [34].

In addition to the purely thermodynamic relationships (1–13), there are some other non purely thermodynamic relationships that can be investigated empirically when the enthalpy and entropy changes upon binding are known. Among them, the enthalpy-entropy compensation phenomena [26] which occur not only in the case of “specific” interactions [12,21] but also in the case of protein adsorption at solid liquid interfaces or in the case of solute binding at fluid interfaces as we will see in the last part of this review.

The enthalpy-entropy compensation seems to be intrinsically associated with the fact that Δ*H*° and Δ*S*° are related to the heat capacity at constant pressure [35].

The enthalpy-entropy compensation expresses according to:

(14) T.ΔS°=β+α.ΔH°

Its validity has been often discussed on the basis of the lack in accuracy in the determination of Δ*H*° and Δ*S*°. However it is clear that if equation (14) holds true, it should have consequences on the thermodynamic Gibbs-Helmholtz relationship (1) which has then to be written as:

(15) ΔG°=(1−α).ΔH°−β

Hence if one finds experimentally that *T*.Δ*S*° is a linear function of the standard enthalpy change with a slope α, one has to check that the free energy change is a linear function of Δ*H*° with the expected slope 1-α. If this consistency test is not satisfied, there is no enthalpy-entropy compensation.

We will not summarize the working principle of modern isothermal titration calorimeters, because this has been extensively done elsewhere [24]. Let us just recall that there are many experimental designs of calorimeters: the hydration calorimeter [36], flow adsorption calorimeters [37,38] which are dedicated to investigate adsorption (often “non specific” phenomena).

The investigation of biophysical and biochemical processes requires the use of highly sensitive calorimeters able to measure heat fluxes of the order of some tens of ncal·s^−1^ with an amount of biomolecules that should be (significantly) less than 1 mg. Indeed, if the standard enthalpy change is of 10 kcal·mol^−1^ (ie 42 kJ·mol^−1^) which is rather typical for biochemical processes, and if the protein concentration is 1 mg/mL (hence about 10^−5^ M (assuming a molecular mass of 10^5^ g·mol^−1^) the total heat exchanged is of the order 10^−4^ cal. If the reaction (or titration) is lasting over 1 h, this means that the average heat flux the microcalorimeter should be able to measure is of the order of 30 ncal·s^−1^. Such sensitivities were not reached for a long time and the major technical evolution was realised only in 1989 [39].

It is not worth herein to describe how to operate such an ITC device nor to describe how to fit a binding model to the experimental data. The interested reader can read the review by O’Brien, Ladbury and Chowdhry [24].

It has to be noted that the last generation ITC devices have now sufficiently fast response to allow the investigation of thermal events occurring during enzymatic catalysis [40–42]. This opens the route for real time studies of a plethora of fascinating biochemical events in real time.

Even if we will occasionally speak about experiments done with differential scanning calorimetry (DSC) and pressure perturbation calorimetry (PPC) we will not describe their working principle. The interested reader can refer to reference [43–45] and [46] for DSC and PPC respectively.

## 3. Some Selected Examples of the Use of ITC to Investigate the Thermodynamics and Kinetics of Biophysical “Unspecific” Processes

According to the previously proposed definition of “non-specific” interactions we will now review the application of ITC (and occasionally of DSC and PPC) to the investigation of the energetics of “non-specific” processes in biophysical chemistry. We will describe phenomena of increasing complexity, where the increasing complexity corresponds to the implication of a greater number of subprocesses. All the investigated process occur at interfaces. We will review the use of microcalorimetry in:

- the interactions between amphiplilic molecules
- the interactions between small ions with polyelectrolytes as well as between polyelectrolytes
- the interactions between drugs, peptides and viruses with lipid assemblies
- the interactions between proteins and solid surfaces.
- the investigation of the global thermal effect of metabolic processes.

All these “non-specific” interactions are of prime importance to biology: let us briefly comment their relevance. The self assembly of lipids allows production of the compartmentalisation which is necessary for the appearance of life [7]. The interaction between charged molecules and single stranded or double stranded DNA is extremely important for the regulation of gene expression since these interactions lead to compaction of DNA [47–50] The same electrostatic interactions are used for the preparation of “complexes” between DNA and cationic species (we put quotes around the word complexes to emphasise that they are not of the same nature as the usual complexes in inorganic chemistry: they do not have a defined stoichiometry). The interaction between a polycation and DNA has to be strong enough to allow efficient DNA overcharging, a *sine qua non* condition for inducing endocytosis of the “complexes” but not to strong in order for the DNA to be liberated from its gene vector in the cytoplasm or in the lyzosome [51]. This means that a deep thermodynamic understanding of polycation-DNA interactions is critical for a rational design of efficient gene vectors.

The interaction between drugs or peptides with biological membranes is at the heart of the transport of amphiphilic drugs across the lipid barrier and the interaction of venoms and viruses with cells [52]. Last but not the least protein adsorption is of tremendous importance in biomaterial science [53] and it most often induces conformational changes of the adsorbed proteins [54]. The investigation of such conformational changes on model surfaces may help to understand the fundamental mechanisms leading to the conformational transition of soluble prions (PrP^C^) to the pathological β sheet rich PrP^SC^ [55,56].

We will not consider the thermodynamics of protein folding-unfolding, because it is a field by itself and because it is a *highly* “specific” process with uses some specific nucleation sites, the so called “molten globule” [57] as well some catalysts (chaperones) [58].

### 3.1. Investigation of the Interaction between Amphiphiles and between Amphiphiles and Polymers

Upon increasing the concentration of amphiphiles (carrying a polar headgroup and at least one hydrophobic tail) in water, after the solution-air interface is saturated with a monolayer, the amphiphiles are forced to be dissolved in water. But the hydrophobic moieties have to be shielded from water to minimise the free energy of the system. Hence the amphiphiles self assemble and there is a critical concentration, called the critical micelle concentration (*cmc*) after which all the added amphiphile is incorporated in the self assembled structures. In the case where the packing ratio, defined as the ratio between the volume of the hydrophobic part and its length multiplied by the area of the hydrophilic head [31], is lower than 1/2, hence when the amphiphile is cone shaped, the self assembled structure is a micelle. When the packing ratio is between 1/2 and 1, which typically occurs for double chained amphiphiles (and hence phospholipids and sphyngolipids), the amphiphiles associate in the form of bilayers. When the packing ratio is greater than one, the stable aggregates are inverted micelles.

At a first order approximation, the value of the *cmc* allows to calculate the change in standard free energy upon micellisation, according to equation (17):

(16) $$\Delta G_{micellisation}^{0}\approx -R.T.\text{ln}(cmc)$$

There are a plethora of experimental techniques allowing to measure the *cmc*, as for instance light scattering, fluorescence spectroscopy, conductometry (applicable only in the case of ionizable head groups) and the use of selective electrodes [59].

Isothermal calorimetry has been used to estimate the enthalpy of micellisation [60,61]. To that aim the amphiphile is solubilized at a concentration above its *cmc* and it is diluted in the cell of the microcalorimeter. It appears that the enthalpies of micellisation are often positive, the process of micelle formation is endothermic. This endothermicity is the balance between several contributions: The association between the hydrocarbon tails (Δ*H_ass_* ≤ 0), their dehydration, (Δ*H_desolvation_* ≥ 0), and the repulsion between the hydrophilic headgroups, (Δ*H_head_* ≥ 0), which is of particular importance in the case of charged amphiphiles. Since the sum of these contributions is endothermic, the repulsion between the headgroups and the desolvation of the hydrophobic tails outweighs the exothermic contribution due to the favourable chain-chain attraction. This is characteristic of the hydrophobic effect.

**Figure 3.**
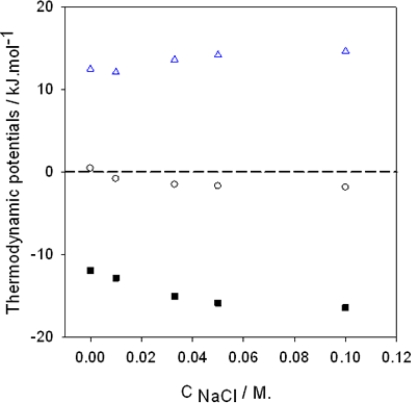
Thermodynamic data describing the micellisation of sodium dodecyl sulfate as a function of the NaCl concentration and at 298 K. Data taken from Refs. [59] and [60]. ( 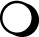): $\Delta H_{\text{micellisation}}^{0}$, ( 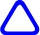): $T.\Delta S_{\text{micellisation}}^{0}$, (▪): $\Delta G_{\text{micellisation}}^{0}$ The dashed line corresponds to a process implying no change in energy.

Nevertheless the micelle formation is a spontaneous process, implying that the entropy change is positive. Again this is expected for a process ruled by hydrophobic interactions [62]. The knowledge of the *cmc* and hence of free energy of micelle formation, equation (16), and the measurement of the enthalpy of micellization, allow to calculate $T.\Delta S_{\text{micellisation}}^{0}$. Figure 3 displays the data taken from the *cmc* measurement of Newberry [59] and the calorimetry data from Birdi [60] in the case of the most used amphiphile, sodium dodecyl sulfate (SDS).

**Figure 4.**
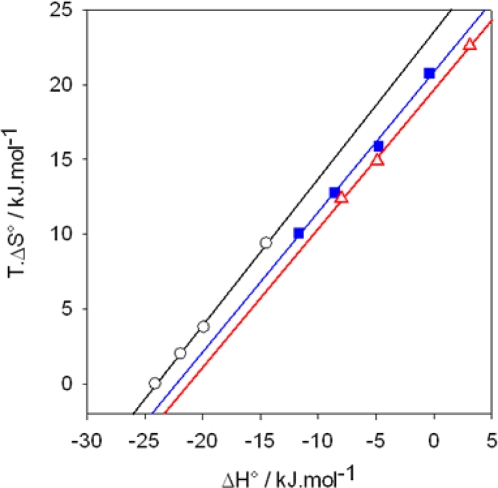
Enthalpy-entropy compensation plots for the micellization of alkylpyridinium surfactants as a function of the used counteranion: ( 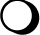): I^−^, the slope of the linear regression is α=0.98, ( 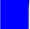): Br^−^, α=0.93, ( 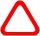): Cl^−^, α=0.93. The ITC experiments were performed between 303 and 333 K. Data taken from Ref. [65].

It appears from Figure 3 that the absolute value of $T.\Delta S_{\text{micellisation}}^{0}$ provides the major contribution to the free energy change. The finding that the micellization process is endothermic is not general, nevertheless the increase in entropy upon micellization is a general observation. There are other studies in the literature dealing with the use of isothermal microcalorimetry to investigate the formation of micelles [61–65]. ITC has been used not only to measure the enthalpy associated with the demicellization process, but also the value of the *cmc* itself [64]. The change in the nature of the counterion of alkylpyridinium surfactants has profound effects on the value of the *cmc* and the change in temperature has effects on the sign of $\Delta H_{\text{micellisation}}^{0}$ (Figure 4) [65]. In addition there is an enthalpy-entropy compensation effect [equation (14)] with a slope α almost equal to 1 whatever the counterion of the alkylpyridinum surfactant. This implies that the free energy change should be a constant (according to equation (15)), which coincides with the experimental finding [65].

ITC has also been employed to investigate the association of amphiphilic polyelectrolytes (like poly-(2-acrylamido)-2-methylpropanesulfonic acid) [66] as well as the interactions of poly(amidoamine) dendrimers with surfactants (the negatively charged SDS as well as cetyltrimethylammonium bromide, CTAB) [67]. This later study is of particular interest because it combines different experimental techniques like ITC, SDS selective electrodes and small angle neutron scattering to investigate the structure of surfactant- dendrimer aggregates.

The thermodynamics of polymer-surfactant vesicles has also been considered from the point of view of phase diagrams. This aspect has been reviewed recently [68].

In the case of double layer forming amphiphiles the energetics of self-association is more complicated to investigate owing to the their very low *cmc* value (typically of the order of 10^−8^ – 10^−9^ M). There are however many DSC investigations aimed to investigate the phase transition from the ordered liquid cristalline to the gel phase transition [69–70]. The hydration thermodynamics of saturated phospholipids (with aliphatic chains carrying 12, 14 or 16 carbon atoms) has been investigation by means of ITC [36]. It has been measured that the association of the 3 first hydration molecules to dehydrated lipids carrying phosphorylcholine head groups is exothermic (typically −16 ± 2 kJ·mol^−1^) whereas the association of further water molecules is endothermic. This endothermicity has been attributed to the water induced melting of the phospholipid from the liquid crystalline to the gel phase.

PPC has been used to investigate the changes in the specific volume of 1,2-dimyristoyl-sn-glycero-3-phosphocholine (DMPC) during its pre-transition and during its main phase transition [71]. The influence of surfactants in the formation of domains in lipid bilayers has also been studied with PPC [72].

ITC has also been used to investigate the effect of osmotic stresses on the area changes of lipid membranes. When water is uptaken by vesicles in contact with an hyper-osmotic solution the vesicles undergo a lateral stretching accompanied by an uptake of thermal energy [73].

### 3.2. Investigation of the Interactions between Small Ions and Polyelectrolytes as well as between Polyelectrolytes

The interaction between charged colloids in aqueous solution is particularly important in biology and its investigation is challenging because of its complexity. The interactions are not purely electrostatic and one has not to forget that each charged colloid or macromolecule is surrounded by a diffuse electrical double layer as well as some strongly bound counterions. Desolvation is also very important, as for amphiphiles. The condensation of counterions to charged macromolecules, which we already mentioned, occurs when its linear charge density exceeds a critical value [10,74]. Such a condensation phenomenon has been observed experimentally for a lot of synthetic polyelectrolytes as well as for DNA. Upon the interaction of two oppositely charged polyelectrolytes, at low salt concentration of the solution (i.e. at low ionic strength) one frequently observes an increase in the conductivity of the solution [75,76] which is due to counterion release. Indeed upon formation of “complexes” between two oppositely charges polyelectrolytes, some of these counterions have to be removed from the contact region between the interacting species. The counterion condensation can be so strong that it can lead to charge inversion of the polyelectrolyte [77,78]. There has been considerable effort to investigate the mechanisms of polyelectrolyte-polyelectrolyte interactions [79] in terms of the influence of polymerisation degree, *pH* in the case of weak polyelectrolytes (among which belong proteins) [80], charge density [81–84], and the presence of low molecular weight electrolytes aimed to screen the electrostatic forces [85]. The structure of such complexes [86–90] as well as their dynamics [82,91–95] has also been intensively investigated. Proton pulsed field gradient NMR has been used to demonstrate that the complexes between poly(diallyldimethylamonium chloride) and bovine serum albumin (BSA) can disintegrate and that dense domains may reform depending on the interaction strength between both partners [91]. Depending on the nature of the polycation used to build complexes with DNA, these complexes can be redisolved by means of an increase in ionic strength [93]. The dissolution efficiency at a given ionic strength depends on the nature of the salt, the most efficient being those containing a cation that strongly interacts with DNA, Ca^2+^ for instance [94]. Even more important is the observation that the DNA may be displaced from the DNA-polycation complexes by means of a polyanion that as a stronger affinity with that polycation than DNA itself [95].

It appears that the most important phenomenological parameter determining the morphology, the charge state and hence the solubility of the complexes as well as their dynamics, is the mixing ratio, *r*, i.e. the ratio between the number of moles of cationic groups and the number of moles of anionic groups. In most (but not) all cases, phase separation between a complex rich and a complex poor phase occurs when *r* is close to 1.

From a theoretical point of view some progress has been made in unravelling the fundamental mechanism of inter-polyelectrolyte complex formation [96–99]. The observation of selectivity effects in the polyanion binding to a given polycation highlights the need to investigate the thermodynamics of the complexation process. The number of investigations aimed to determine the binding constant of the polyelectrolyte-ion or polyelectrolyte-polyelectrolyte complexes are pretty scarce [95,100–104]. Among the most efficient methods to directly measure binding constant are fluorescence quenching methods: Lohman’s group investigated the interactions between positively charged lysine oligopeptides modified with a tryptophan residue and various oligonucleotides. The binding event causes a total quenching of the tryptophan fluorescence emission [102]. In the case of complexes implying DNA it is possible to use the fluorescence emission properties of ethidium bromide (EtBr): In the DNA intercalated state it is strongly fluorescent but upon complexation with a polycation, EtBr is removed from DNA and its fluorescence quantum yield is dramatically reduced [93,95].

The binding isotherms obtained give the amount of bound molecules to their binding partner as a function of the concentration of unbound molecules. These isotherms have to be fitted to a binding model [2,105–107] which yields the equilibrium constant or a set of microscopic equilibrium constants in the case of cooperative interactions. Before the use of microcalorimetry, van’t Hoff analysis of the temperature dependence of the equilibrium constant(s) was used to estimate the enthalpy change upon binding.

As in the case of interactions between amphiphiles, there are many sub-processes contributing to the enthalpy and entropy changes: hydration changes, changes in the conformation of the polyelectrolytes and of course electrostatic effects. Owing to the presence of counterions around the polyelectrolytes, these electrostatic interactions are always the result of a competition between the counterions bound to each partner before their interaction and the electrostatic bonds formed in the final state. Indeed, this competition is used in the fluorescence quenching assays using EtBr [93,95]. The importance of counterion release to the free energy change upon polyelectrolyte-polyelectrolyte binding can be estimated by studying the influence of ionic strength: its increase produces a decrease in affinity not only because the electrostatic interactions between the polyelectrolytes are screened but also because of entropic effects [108].

**Figure 5.**
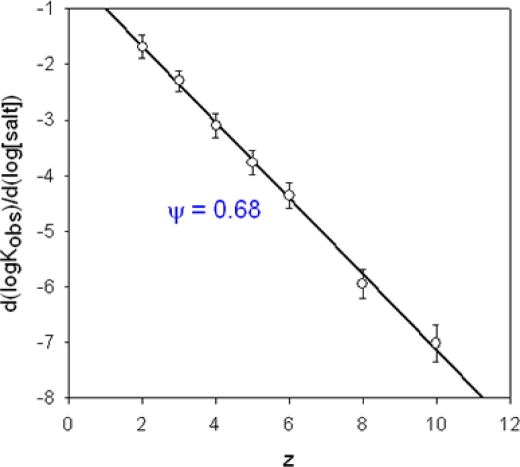
Variation of the logarithm of the Trp-(Lys)_n_ (2 ≤ *n* ≤ 8)-polyU binding constant as a function of the valence of the polypeptide. The slope of the straight line yields ψ=0.68, according to equation (17). Data from Table 1 of reference [109].

The dependence of the binding constant with respect to the concentration of the supporting electrolyte is given by:

(17) $$\frac{\partial (\text{log}K)}{\partial C_{electrolyte}}=-z.\psi$$

where *z* is the valence of the interacting polyelectrolyte with an oppositely charged binding partner, the “receptor” (of higher valency), and ψ is the average number of counterions released by a charged group of the “receptor” polyelectrolyte. A general derivation of equation [17] can be found on pages 871–873 of reference [2]. An example of such an analysis of the binding data between a series of oligolysines (modified with a single tryptophan residue to monitor fluorescence quenching) and single-stranded homopolynucleotides (polyU) is displayed in Figure 5 [109]. This plot shows that on average 7 potassium counterions are lost by every 10 phosphate group of polyU upon its binding with Trp-(Lys)_n_ (2 ≤ *n* ≤ 8).

Additional examples of such counterion release phenomena can be found in the literature [110–112]. In most of these studies, the enthalpy and entropy changes have been calculated from a van’t Hoff analysis of the data [equation (11)] with some exceptions however, where the enthalpy changes where measured directly by means of ITC [111]. When changing the NaCl concentration, the enthalpy change associated with the single-stranded binding tetramer and (polyT)_69_ follows the following law [111]:

(18) $$\Delta H_{\text{obs}}=\alpha .\text{log}\left[NaCl\right]+\beta$$

with: *α* = 46.3±1.8 kcal.mol^−1^ and *β* = −109.8±0.9 kcal.mol^−1^.

**Figure 6.**
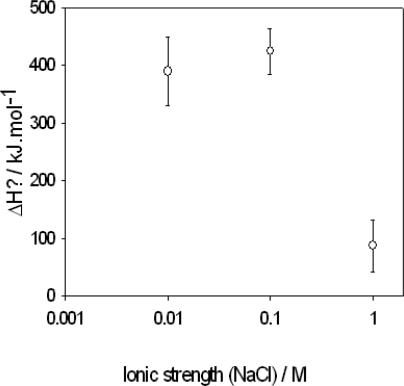
Variation of the binding enthalpy between BSA and PAH as a function of the ionic strength, as obtained from ITC [113].

These experimental data are in reasonable agreement with those obtained from a van’t Hoff analysis but not identical [111]. At low ionic strength, the interaction enthalpy is exothermic and decreases in absolute value when the ionic strength increases. The same trend has been observed for the interactions between bovine serum albumin (BSA) and poly(allylamine) (PAH) in conditions where the protein and the polycation are oppositely charged (10 mM Tris buffer, *x* M NaCl at *pH* 7.4) [113], Figure 6. In this case, the interaction enthalpy was *endothermic*, which clearly shows that the BSA-PAH complexation process is driven by an increase in entropy. Unfortunately, a whole titration curve could not be obtained by means of ITC because of phase separation phenomena occurring upon an increase in the BSA/PAH ratio. This seems to be a recurrent difficulty in the investigation of polyelectrolyte–polyelectrolyte complexation, even if some data are available [114–116]. It appears that the inter-polyelectrolyte complexation process is driven through an increase in entropy, as was already suggested very early in the literature [117]. In addition, the ITC binding curves have been interpreted *with a very simple and unrealistic binding model*: a one to one stoichiometry between anionic and cationic sites. This model totally neglects cooperative binding effects as well as a possible mismatch between the cationic and anionic sites. From this point of view it seems urgent to perform ITC titrations as well as measurement of the binding constant by an independent method (for instance fluorescence quenching) in the case of monodisperse polyelectrolytes of variable charge density. Charged homopolypeptides (cationic: (Lys)_n_, anionic (Glu)_n_ or (Asp)_n_) as well as aliphatic *n,n*-ionenes [93] seem to be very promising candidates to achieve this aim.

For a whole set of polycation-polyanion combinations it has been found that the enthalpy change upon complexation turns from exothermic to endothermic upon an increase in salt concentration [118]. These data have been obtained from ITC in the case where the mixing ratio was small (hence assuming that all the injected moles of anions find a positively charged binding partner). This trend was correlated with a change in the growth regime of layer-by-layer polyelectrolyte multilayer films from a linear growth at low salt concentration to an “exponential growth” at higher salt concentration. The polyelectrolyte multilayer films do not growth anymore above a critical ionic strength [118]. This observation may be correlated with the dynamics of the interpolyelectrolyte complexes as recently suggested by Cohen Stuart *et al*. [92]: the complexes seem to be in a frozen state at low salt concentration and highly hydrated and flexible at high salt concentration.

The interactions between synthetic polyelectrolytes and water [119], as well as the interactions between DNA and ions [120–122] or between proteins and ions have been investigated by means of ITC [123,124].

**Figure 7.**
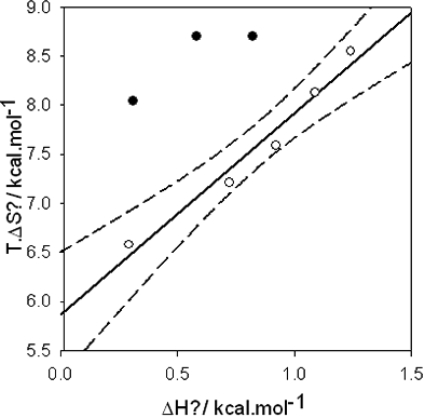
Enthalpy-entropy compensation plot for the ITC data of Bloomfiled *et al*. [121] describing the two interaction regimes between $Co(NH_{3}{)}_{6}^{3+}$ and DNA. Enthalpy and entropy changes associated with: ( 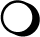) $Co(NH_{3}{)}_{6}^{3+}$-pUC118 binding and: (•) condensation of pUC118. The full and dashed lines correspond to the linear regression and the limit of the 95 % confidence interval respectively.

The dilution heat of polystyrene sulfonate [119] as well as of other polyelectrolytes [118,123] is endothermic as expected from the counterion condensation theory [10]. The interaction between multivalent ions like cobalt hexaamine and circular pUC118 DNA displays a biphasic behavior, both events being endothermic. The first one is associated with the binding of $Co(NH_{3}{)}_{6}^{3+}$ to isolated phosphate groups whereas the second one is associated with the cation binding induced condensation of DNA. The transition from the first to the second binding regime is shifted to higher $\frac{Co{\left(NH_{3}\right)}_{6}^{3+}}{\text{phosphate}}$ ratios when the NaCl concentration is increased. This reflects again a competition between Na^+^ and $Co(NH_{3}{)}_{6}^{3+}$ for the phosphate groups of DNA [121]. Most interestingly, the trivalent spermidine cations are less efficient than $Co(NH_{3}{)}_{6}^{3+}$ to induce condensation of DNA, which is expected on the basis of the higher charge density of $Co(NH_{3}{)}_{6}^{3+}$. Of additional interest is the fact that the first binding event displays an enthalpy-entropy compensation phenomenon whereas this seems to be hardly the case for the second one (Figure 7). The same kind of biphasic titration has been found during the investigation of the interactions between cetyltrimethylammonium bromide and DNA [122].

We will now shift to the description of the use of ITC to the investigation of drug-lipid assemblies interactions. We will also describe the interactions between charged molecules (among which polypeptides, polyelectrolytes and viruses) and lipid assemblies.

### 3.3. Investigation of the Interactions between Drugs, Peptides and Viruses with Lipid Assemblies

**Figure 8.**
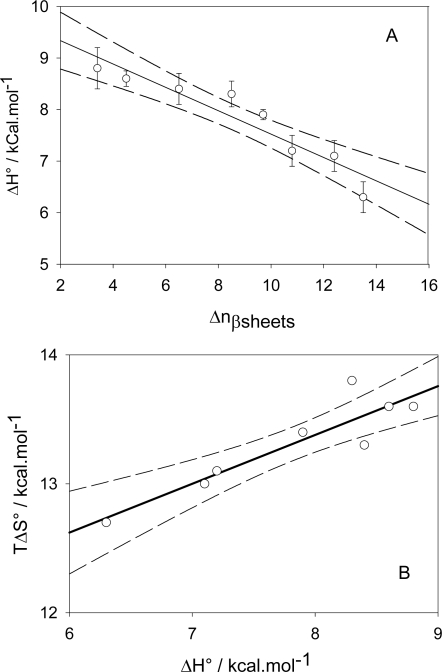
Thermodynamic data describing the interactions between (KIGAKI)_3_ peptides (in 5 mM cacodylate buffer at *pH* 7.5) and small unilamellar vesicles made of POPE/POPG/pegylated POPE (70/25/5 mol%). Part A: relation between the enthalpy change (recorded from an ITC titration) and the increase in the number of amino acids implied in β sheet structures (obtained from CD spectroscopy). The full line corresponds to a linear regression to the data (r^2^=0.89) whereas the dashed line corresponds to the limit of the 95 % confidence interval. Part B: Enthalpy-entropy compensation plot corresponding to the same peptide/vesicle system. The full line corresponds to a linear regression to the data (r^2^=0.83) whereas the dashed line corresponds to the limit of the 95% confidence interval. Data taken from Ref. [137].

Less attention will be given on these kinds of interactions than on those occurring upon inter-polyectrolyte or small ions-polyelectrolyte interactions, because very good reviews are already available [124–126] in addition to a plethora of research articles [127–141]. The interactions taking place at the surface of lipid membranes imply not only electrostatic interactions, of the same nature as those described in the previous paragraph, but also some conformational changes, as well as some partioning events between the aqueous phase and the membrane phase, and eventually some membrane fusion events. NMR in addition to circular dichroïsm (CD) investigations will allow to estimate the contribution of conformational changes to the binding process [135–137]. In the case of (KIGAKI)_3_ polypeptides (K, I, G and A are the one letter representation of lysine, isoleucine, glycine and alanine respectively) having some l-enantiomers of alanine substituted by d-enantiomers and interacting with POPE/POPG/pegylated POPE (70/25/5 mol%) vesicles, it has been found that the peptides bound to the vesicles undergo a progressive conformational transition to β sheets. CD has been used for this aim. The enthalpy of binding is endothermic, meaning that the binding process is driven by a global increase in entropy and Δ*H*° is directly proportional to the increase in the number of amino acids that become incoporated in a β sheet structure (Figure 8) [137].

In general, the contribution from electrostatic interactions and from hydrophobic interactions can be separated when the charge state of the binding molecule is known, using for instance a modified form of the Gouy-Chapman theory [142]. In a very interesting investigation, it has been demonstrated that the interaction between magainin-2-amide with membranes made from 1-palmitoyl-2-oleoyl-sn-glycero-3-phosphocholine (POPC) (75 mol%) and 1-palmitoyl-2-oleoyl-sn-glycero-3-phophoglycerol (POPG) displays a biphasic trace: at low peptide to lipid ratios, the enthalmograms obtained by ITC reflect the peptide binding process (−17 ± 1 kcal·mol^−1^) whereas at high peptide to lipid ratios an endothermic process (6.2 ± 1.6 kcal·mol^−1^) is due to the peptide induced pore formation. ITC has also been used to investigate the fusion of the Influenza virus with vesicles and a biphasic binding process was also monitored [145].

Very interestingly there are some drug-vesicle interactions that are of purely “specific nature” [133].

**Figure 9.**
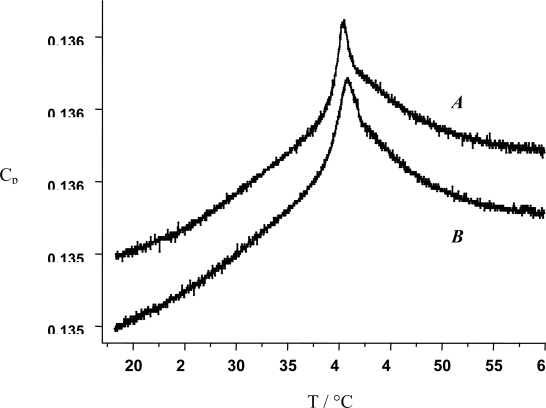
DSC traces of large unilamellar vesicles made from a DPPC/DPPG/CL mixture (80/10/10 w/w) in the absence (A) and in the presence of adsorbed PLL (B). Modified from reference [144]. *Cp* is given in kCal.mol^−1^.K^−1^.

DSC is also very useful to estimate the conformational destabilization of proteins upon their interaction with membranes [138] as well as to answer the question if polyelectrolytes interact with charged membranes with a perturbation [143] or not of the membrane integrity [144]. The former phenomenon seems to occur when the polyelectrolyte carries hydrophobic side chains whereas the later occurs for non modified poly-l-Lysine (PLL) interacting with large unilamellar vesicles made from a DPPC/DPPG/CL mixture (80/10/10 w/w) [144]. In this case the DSC trace of the PLL decorated vesicles is identical (Figure 9) to that of the pure dipalmytoyl phosphatidylcholine/dipalmytoyl phosphatidylglycerol/cholesterol (CL) mixture, reflecting that the main phase transition of the lipid mixture (here at 41 °C) is not modified by the presence of the adsorbed PLL (charge inversion of the vesicles upon PLL adsorption has been demonstrated by means of zeta potential measurements).

### 3.4. Investigation of the Interactions between Proteins and Solid Surfaces

As already mentioned, the interactions between proteins and solid surfaces are extremely important in modern technology, for instance in fouling processes and in biomaterials science. Chromatographic analysis of amino acid retention on solid supports has allowed investigation of the adsorption behaviour of single amino acids on solid surfaces [146], but extrapolation to polypeptides and to proteins is difficult because of the complexity of the process. Major advances have been made in the understanding of this very ubiquitous phenomenon by Norde [147–149]. ITC as well as DSC played a major contribution in this research. It was found that proteins can be classified mostly into two categories with respect to their adsorption behaviour at solid/liquid interfaces: the classification between “hard” and “soft” proteins refers to the difference in free energy between the unfolded and folded conformations [147]. In general “soft” proteins are able to adsorb at any kind of sorbent even if it is hydrophilic and electrostatically repulsive, whereas “hard” proteins, having a high conformational stability, only adsorb at hydrophilic surfaces in the case of favourable electrostatic interactions. At hydrophobic surfaces, dehydration of that surface is usually the dominant driving force for adsorption, so that both “hard” and “soft” proteins adsorb, even under electrostatically adverse conditions. Clearly, DSC is extremely useful to distinguish between “hard” and “soft” proteins. There are many examples in which protein adsorption at solid/liquid interfaces is driven through an increase in entropy. Dehydration of the sorbent and of the protein surface (which is never purely hydrophilic, [147]) as well as partial protein unfolding are at the origin of this entropy increase. The combination between DSC and infra red spectroscopy [150] as well as between IR spectroscopy and hydrogen-deuterium exchange mass spectrometry [151] allows to directly correlate the thermodynamic decrease in protein stability at the surface to the change in its average conformation.

Recently, much effort has been devoted to the investigation of protein adsorption on the stationary phases of chromatographic columns [152–156]. Even if the enthalpy changes upon adsorption have been directly measured in some cases, most of the investigations rely on the fit of an adsorption model to the adsorption isotherm. This was done to calculate the adsorption equilibrium constant. Such purely empirical fitting of the adsorption isotherms, using for instance the Langmuir isotherm may be very arbitrary in the sense that this model totally neglects the interactions between the adsorbed proteins (see reference [17] and references therein).

**Figure 10.**
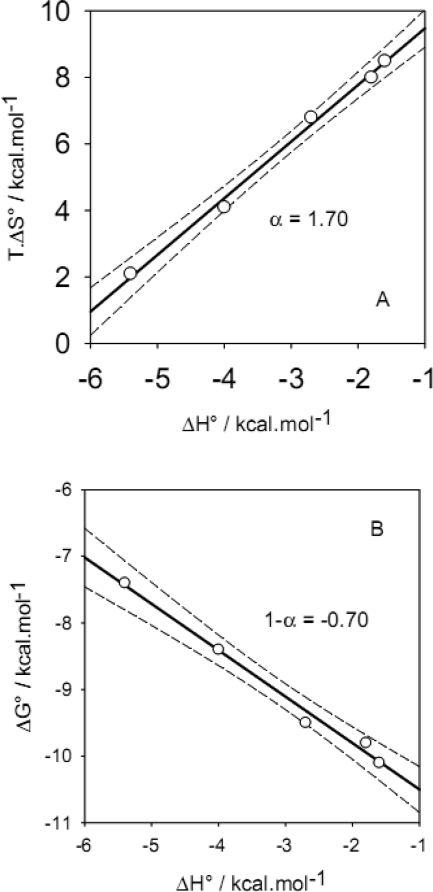
A: Enthalpy-entropy compensation plot for the adsorption of statherin onto hydroxyapatite particles (having a specific surface area of 53 m^2^.g^−1^) at different temperatures (between 15 and 37°C) in the presence of phosphate buffer. Data from Table 1 of reference [157]. The slope of the linear regression curve (r^2^=0.995) is α=1.70, calculated according to equation (14). B: correlation between the change in standard free energy upon adsorption with the enthalpy change. The slope of the linear regression curve (r^2^=0.989) is equal to −0.70 in full agreement with the α value obtained in part A and with equation (15), showing that there is a “true” enthalpy-entropy compensation. The dashed lines represent the limits of the 95 % confidence intervals.

ITC data of protein adsorption at colloid particles have also revealed enthalpy-entropy compensation relationships [157–158], Figure 10. It has been demonstrated by performing the ITC experiments in buffers having different ionisation enthalpies (phosphate, Tris and TES buffer) that every single protein adsorption event is accompanied by the incorporation of one proton in the adsorbed layer [157].

With the aid of ITC it has been demonstrated recently that the interaction between salivary proteins and *Streptococcus Mutans* is a non specific process when the microorganism is depleted of its specific surface antigens [159].

### 3.5. Investigation of the Global Thermal Effect of Metabolic Processes

Very interestingly, microcalorimetry has been used to investigate the heat exchanges implied in extremely complicated processes such as the metabolisms of living cells [161,162]. It is possible to distinguish the exponential growth phase of cells from the initial lag phase with a single microcalorimetry measurement. This offers the opportunity to compare the metabolism of different cell lines, and possibly the difference between health cells and cells implied in a pathological process.

## 4. Conclusions and Perspectives

In this review article we have recalled the difference between “specific” and “non-specific interactions” and have emphasized how difficult it is to distinguish between both interaction modes. Based on many data from the biophysical and biochemical literature (as well as from supramolecular chemistry) it appears possible that many interactions have contributions coming from “non-specific” interactions. These “non-specific” contributions originate mostly from electrostatic and hydrophobic interactions. We have then shown that ITC (as well as DSC) gives reliable enthalpy and entropy changes of “non-specific” binding events. We focused on interactions between amphiphiles, amphiphiles and polymers, polyelectrolyte-polyelectrolye, polyelectrolyte-small electrolytes, molecules (drugs, peptides) and lipid membranes and proteins with surfaces. It appears that the field of “ligand” binding at membrane/aqueous solution interfaces is the most mature of these fields in the sense that it is possible to unravel different contributions to the global enthalpy and entropy changes. The complete understanding of the interactions between polyelectrolytes is challenging in order to improve the efficiency of gene transfection and surface coating by the layer-by-layer deposition. It appears that even if ITC provides now accurate values of enthalpy changes, it will be extremely difficult to quantify the (positive) entropy changes upon complexation owing to the fact that phase separation occurs when the inter-polyelectrolyte complexes become electroneutral. Hence the determination of binding constants by traditional titration methods, for instance fluorescence spectroscopy, coupled with ITC measurement of enthalpy changes will allow to gain better understanding in the thermodynamics of such fascinating (but ill defined) processes. It is also mandatory to investigate complexation processes between extremely well characterised and monodisperse polyelectrolytes to unravel the contributions from counterion release and those from conformational changes upon inter-polyelectrolyte complexation. The combination of purely experimental approaches with simulations (Monte Carlo as well as molecular dynamics) will also be a source of progress in understanding the “non-specific” interactions between polyelectrolyes. In all the investigated cases, interesting enthalpy-entropy compensations were observed. Some more research is needed to understand their origin.

## References

[1] Ruben AJ Kiso Y Freire E Overcomming roadblocks in lead optimization:a thermodynamic perspective Chem. Biol. Drug Des 2006 67 2 4 16492143 10.1111/j.1747-0285.2005.00314.x

[2] Cantor CR Shimmel PR Biophysical Chemistry. Part III. The behavior of biological macromolecules W.H. Freeman and Company New York, NY, USA 1980 Chapter 15.

[3] Rose T di Cera E Thermodynamic dissection of cooperativity in ligand recognition Thermodynamics in Biology Oxford University Press New York, NY, USA 2000 Chapter 2.

[4] Lehn JM Supramolecumar Chemistry Wiley-VCH. Weinheim Berlin, Germany 1995

[5] Alberts B Bray D Lewis J Kaff M Roberts K Watson JD Molecular Biology of the Cell3rd Ed Garland Publishing Inc New York, NY, USA 1993

[6] Lyklema J Quest for ion-ion correlations in electric double layers and overcharging phenomena Adv Colloid Interface Sci 2009 147–148 205 213 10.1016/j.cis.2008.12.002 19162256

[7] Lambert J-F Adsorption and polymerization of amino acids on mineral surfaces: a review Origins Life Evol. Biosphere 2008 38 211 242 10.1007/s11084-008-9128-3 18344011

[8] Qiu T Barteau MA STM study of glycine on TiO_2_ (110) single crystal surfaces J. Colloid Interface Sci 2007 303 229 235 16919666 10.1016/j.jcis.2006.07.053

[9] Lyklema J Electrical double layer on silver iodide. Influence of temperature and application to sol stability Discuss. Faraday Soc 1966 42 81 90

[10] Manning GSQ The molecular theory of polyelectrolyte solutions with applications to the electrostatic properties of polynucleotides Rev. Biophys 1978 11 179 246 10.1017/s0033583500002031 353876

[11] Kunz W Lo Nostro P Ninham BW The present state of affairs with Hofmeister effects Curr. Opin. Colloid Interface Sci 2004 9 1 18

[12] Houk KN Leach AG Kim SP Zhang X Binding affinities of host-guest, protein-ligand, and protein-transition state complexes Angew. Chem. Int. Ed 2003 42 4872 4897 10.1002/anie.200200565 14579432

[13] Jones S Thornton JM Principles of protein-protein interactions Proc. Natl. Acad. Sci 1996 93 13 20 8552589 10.1073/pnas.93.1.13 PMC40170

[14] Gerschel A Liaisons intermoléculaires. Les forces mises en jeu dans la matière condensée Inter Editions/CNRS Editions Ed Paris, France 1995

[15] Ball V Adsorption behaviour of different polypeptides in the 3 kDa molecular weight range at an Si_0.8_Ti_0.2_ O_2_-aqueous solution interface from low ionic strength solution Colloids Surf., B: Biointerfaces 2004 33 129 142

[16] Latour RA Molecular simulation of protein-surface interactions: Benefits, problems, solutions, and future directions Biointerphases 2008 3 FC2 FC12 19809597 10.1116/1.2965132 PMC2756768

[17] Schaaf P Voegel J-C Senger B From random sequential adsorption to ballistic deposition: a general view of irreversible deposition processes J. Phys. Chem. B 2000 104 2204 2214

[18] Smith R Tanford C Hydrophobicity of long chain n-alkyl carboxylic acids, as measured by their distribution between heptane and aqueous solutions Proc. Natl. Acad. Sci. USA 1973 70 289 293 16592052 10.1073/pnas.70.2.289 PMC433241

[19] Hamacek J Borkovec M Piguet C Simple thermodynamics for unravelling sophisticated self-assembly processes Dalton Trans 2006 12 1473 1490 16538265 10.1039/b518461d

[20] Rekharsky MV Inoue Y Complexation thermodynamics of cyclodextrins Chem Rev 1998 98 1875 1918 11848952 10.1021/cr970015o

[21] Jelesarov I Bosshard HR Isothermal titration calorimetry and differential scanning calorimetry as complementary tools to investigate the energetics of biomolecular recognition J. Mol. Recognit 1999 12 3 18 10398392 10.1002/(SICI)1099-1352(199901/02)12:1<3::AID-JMR441>3.0.CO;2-6

[22] Harding SE Chowdry BZ Protein-ligand interactions: hydrodynamics and calorimetry Practical Approach Series, Oxford University Press New York, NY, USA 2001 Chapters 2-7.

[23] Rogniaux H Sanglier S Strupat K Azza S Roitel O Ball V Tritsch D Branlant G van Dorsselaer A Mass spectrometry as a novel approach to probe cooperativity in multimeric enzymatic systems Anal. Biochem 2001 291 48 61 11262155 10.1006/abio.2000.4975

[24] O’Brien R Ladbury JE Chowdhry BZ Isothermal titration calorimetry of biomolecules “Protein-ligand interactions: hydrodynamics and calorimetry” Practical Approach Series; Harding SE Chowdhry BZ Oxford University Press New York, NY, USA 2001

[25] Schuck P Use of surface plasmon resonance to probe the equilibrium and dynamic aspects of interactions between biological macromolecules Annu. Rev. Biophys. Biomol. Struct 1997 26 541 566 9241429 10.1146/annurev.biophys.26.1.541

[26] Winzor DJ Jackson CM Interpretation of the temperature dependence of equilibrium and rate constants J. Mol. Recognit 2006 19 389 407 16897812 10.1002/jmr.799

[27] Liu Y Sturtevant JM Significant discrepancies between van’t Hoff and calorimetric enthalpies Protein Sci 1995 4 2559 2561 8580846 10.1002/pro.5560041212 PMC2143031

[28] Pfeil W Privalov PL Thermodynamic investigations of proteins. II. Calorimetric study of lysozyme denatured by guanidinium hydrochmoride Biophys. Chem 1976 4 33 40 1247649 10.1016/0301-4622(76)80004-x

[29] Privalov PL Tiktopulo EI Venyaminov SY Griko YV Makhatadze GI Khechinashvili NN Heat capacity and conformation of proteins in the denatured state J. Mol. Biol 1989 205 737 750 2538636 10.1016/0022-2836(89)90318-5

[30] Kauzmann W Some factors in the interpretation of protein denaturation Adv. Protein Chem 1959 14 1 63 14404936 10.1016/s0065-3233(08)60608-7

[31] Israelachvili J Intermolecular and Surface forces Academic Press San Diego, CA, USA 1991

[32] Tanford C The hydrophobic effect: formation of micelles and biological membranes2nd ed Krieger Publishing Company Malabar, FL, USA 1991

[33] Shimokhina N Bronowska A Homans SW Contribution of ligand desolvation to binding thermodynamics in a ligand-protein interaction Angew. Chem. Int. Ed 2006 45 6374 6376 10.1002/anie.200602227 16906619

[34] Bingham RJ Findlay JBC Hsieh SY Kalverda AP Kjellberg A Perrazolo C Phillips SEV Seshadry K Trinh CH Turnbull WB Bodenhausen G Homans SW Thermodynamics of binding of 2-methoxy-3-isopropylpirazine and 2-methoxy-3-isobutyl pirazine to the major urinary protein J. Am. Chem. Soc 2006 126 1675 1681 14871097 10.1021/ja038461i

[35] Lumry R Uses of enthalpy-entropy compensation in protein research Biophys. Chem 2003 105 545 557 14499917 10.1016/s0301-4622(03)00065-6

[36] Markova N Sparr E Wadsö L Wennerström H A calorimetric study of phospholipid hydration. Simultaneous monitoring of enthalpy and free energy J. Phys. Chem. B 2000 104 8053 8060

[37] Groszek AJ Flow adsorption microcalorimetry Thermochim. Acta 1998 312 133 143

[38] Fubini B Adsorption calorimetry in surface chemistry Thermochim. Acta 1988 135 19 29

[39] Wiseman T Williston S Brandts J-F Lin L-N Rapid measurement of binding constants and heats of binding using a new titration calorimeter Anal. Biochem 1989 179 131 137 2757186 10.1016/0003-2697(89)90213-3

[40] Morin PE Freire E Direct calorimetric analysis of the enzymic activity of yeast cytochrome c oxydase Biochemistry 1991 30 8494 8500 1653014 10.1021/bi00098a030

[41] Todd MJ Gomez J Enzyme kinetics determined using calorimetry: a general assay for enzyme activity? Anal. Biochem 2001 296 179 187 11554713 10.1006/abio.2001.5218

[42] Henzler K Haupt B Ballauff M Enzymatic activity of immobilized enzyme determined by isothermal titration calorimetry Anal. Biochem 2008 378 184 189 18440294 10.1016/j.ab.2008.04.011

[43] Privalov PL Potekhin SA Scanning microcalorimetry in studying temperature-induced changes in proteins Methods Enzymol 1986 131 4 51 3773768 10.1016/0076-6879(86)31033-4

[44] Freire E Differential scanning calorimetry Methods Mol. Biol 1995 40 191 218 7633523 10.1385/0-89603-301-5:191

[45] Johnson CM Differential scanning calorimetry: theory and practice MicroCal Application note.

[46] Lin LN Brandts JF Brandts JM Plotnikov V Determination of the volumetric properties of proteins and other solutes using pressure perturbation calorimetry Anal. Biochem 2002 302 144 160 11846388 10.1006/abio.2001.5524

[47] Gosule LC Schellman JA Compact form of DNA induced by spermidine Nature 1976 259 333 335 1250371 10.1038/259333a0

[48] Wilson RW Bloomfield VA Counterion-induced condensation of desoxyribonucleic acid: a light scattering study Biochemistry 1978 18 2192 2196 444448 10.1021/bi00578a009

[49] Bloomfield VA DNA condensation by multivalent cations Biopolymers 1998 44 269 282 9591479 10.1002/(SICI)1097-0282(1997)44:3<269::AID-BIP6>3.0.CO;2-T

[50] Raspaud E Delacruz MO Sikorav JL Livolant F Precipitation of DNA by polyamines-a polyelectrolyte behavior Biophys. J 1998 74 381 393 9449338 10.1016/S0006-3495(98)77795-1 PMC1299390

[51] Schaffer DV Fidelman NA Dan N Lauffenburger DA Vector unpacking as a potential barrier for receptor-mediated polyplex gene delivery Biotechnol. Bioeng 2000 67 598 606 10649234 10.1002/(sici)1097-0290(20000305)67:5<598::aid-bit10>3.0.co;2-g

[52] Tosteson MT Holmes SJ Razin M Tosteson DC Mellitin lysis of red cells J. Membr. Biol 1985 87 35 44 4057243 10.1007/BF01870697

[53] Llanos GR Sefton MV Heparin-poly(ethylene glycol)-poly(vinyl alcohol) hydrogel: preparation and assessment of thrombogenicity Biomaterials 1992 13 421 424 1633215 10.1016/0142-9612(92)90161-g

[54] Haynes CA Norde W Structures and stabilities of adsorbed proteins J. Colloid Interface Sci 1995 169 313 328

[55] Noinville S Chich JF Rezaei H Misfolding of the prion protein: linking biophysical and biological approaches Vet. Res 2008 39 48 18533092 10.1051/vetres:2008025

[56] Stefani M Prorein Folding and misfolding on surfaces Int. J. Mol. Sci 2008 9 2515 2542 19330090 10.3390/ijms9122515 PMC2635651

[57] Dobson CM Protein folding and misfolding Nature 2003 426 884 891 14685248 10.1038/nature02261

[58] Hartl FU Hayer-Hartl M Molecular chaperones in the cytosol: from nascent chain to folded protein Science 2002 295 1852 1858 11884745 10.1126/science.1068408

[59] Newbery JE The variation of the critical micelle concentration of sodium dodecyl sulphate with ionic strength monitored by selective-ion membrane electrodes Colloid Polym. Sci 1979 257 773 775

[60] Birdi KS Calorimetric determination of the enthalpy of micelle formation in aqueous media Colloid Polym. Sci 1983 261 45 48

[61] Spink CH Chaires JB Thermodynamics of the binding of a cationic lipid to DNA J. Am. Chem. Soc 1997 119 10920 10928

[62] Blokzijl W Engberts JBFN Hydrophobic effects. Opinion and facts Angew. Chem. Int. Ed 1993 32 1545 1579

[63] Bijma K Engberts JBFN Haandrikman G van Os N Blandamer MJ Butt MD Cullis PM Last PM Thermodynamics of micelle formation by 1-methyl-4-alkylpyridinium halides Langmuir 1994 10 2578 2582

[64] van Os N Daane GJ Haandrikman G The effect of chemical structure upon the thermodynamics of micellization of model alkylarenesulfonates: III. Determination of the critical micelle concentration and the enthalpy of demicellization by means of microcalorimetry and a comparison with the phase separation model J. Colloid Interface Sci 1991 141 199 217

[65] de Gooijer JM Engberts JBFN Blandamer MJ A titration microcalorimetric study of the effects of halide counterions on vesicle-forming aggregation in aqueous solution of branched chain alkylpyridinium surfactants J. Colloid Interface Sci 2000 224 4 10 10708488 10.1006/jcis.1999.6668

[66] Bangar Raju B Winnik FM Morishima Y A look at the thermodynamics of the association of amphiphilic polyelectrolytes in aqueous solutions: strenghts and limitations of isothermal titration calorimetry Langmuir 2001 17 4416 4421

[67] Sidhu J Bloor DM Couderc-Azouani S Penfold J Holzwarth JF Wyn-Jones E Interactions of Poly(amidoamine) dendrimers with the surfactants SDS, DTAB, and C_12_EO_6_: An equilibrium and structural study using a SDS selective electrode, isothermal titration calorimetry, and small angle neutron scattering Langmuir 2004 20 9320 9328 15461524 10.1021/la0494932

[68] Antunes FE Marques EF Miguel MG Lindman B Polymer-vesicle association Adv Colloid Interface Sci 2009 147–148 18 35 10.1016/j.cis.2008.10.001 19058777

[69] Ladbrooke BD Chapman D Thermal analysis of lipids, proteins, and biological membranes: A review ans summary of some recent studies Chem. Phys. Lipids 1969 3 304 356 4905514 10.1016/0009-3084(69)90040-1

[70] Chapman D Urbina J Biomembrane phase transitions. Studies of lipid-water systems using differential scanning calorimetry J. Biol. Chem 1974 249 2512 2521 4132554

[71] Heerklotz H Seelig J Application of Pressure Perturbation Calorimetry to Lipid Bilayers Biophys. J 2002 82 1445 1452 11867459 10.1016/S0006-3495(02)75498-2 PMC1301945

[72] Heerklotz H Triton promotes domain formation in Lipid raft mixtures Biophys. J 2002 83 2693 2701 12414701 10.1016/S0006-3495(02)75278-8 PMC1302353

[73] Nebel S Ganz P Seelig J Heat changes in lipid membranes under sudden osmotic stress Biochemistry 1997 36 2853 2859 9062114 10.1021/bi961839n

[74] Manning GS Limiting laws and counterion condensation in polyelectrolyte solutions. I Colligative properties J. Chem.Phys 1969 51 924 933

[75] Acar N Tulun T Studies on the interaction of poly(4-vinypyridinium chloride) with poly(sodium phosphate) in an aqueous solution by conductometry J. Polym. Sci. A. Polym.Chem 1996 34 1251 1260

[76] Webster L Huglin MB Robb ID Complex formation between polyelectrolytes in dilute aqueous solution Polymer 1997 38 1373 1380

[77] Nguyen TT Grosberg AY Shklovskii BI Macroions in salty water with multivalent ions: giant inversion of charge Phys. Rev. Lett 2000 85 1568 1571 10970556 10.1103/PhysRevLett.85.1568

[78] Kleimann J Gehin-Deval C Auweter H Borkovec M Super stoechiometric neutralization in particle – polyelectrolyte systems Langmuir 2005 21 3688 3698 15807622 10.1021/la046911u

[79] de Kruif CG Weinbreck F de Vries R Complex coacervation of proteins and anionic polysaccharides Curr. Opin. Colloid Interface Sci 2004 9 340 349

[80] Hallberg RK Dubin PL Effect of *pH* on the binding of β-Lactoglobulin to sodium polystyrene sulfonate J. Phys. Chem. B 1998 102 8629 8633

[81] Dautzenberg H Jaeger W Effect of charge density on the formation and salt stability of polyelectrolyte complexes Macromol. Rapid Commun 2002 203 2095 2102

[82] Trukhanova ES Izumrudov VA Litmanovich AA Zelikin AN Recognition and selective binding of DNA by ionenes of different charge density Biomacromolecules 2005 6 3198 3201 16283746 10.1021/bm050536i

[83] Mattison KW Dubin PL Brittain IJ Complex formation between bovine serum albumin and strong polyelectrolytes: effect of polymer charge density J. Phys. Chem. B 1998 102 3830 3836

[84] Strand SP Danielsen S Christensen BE Vårum KM Influence of chitosan structure on the formation and stability of DNA-chitosan polyelectrolyte complexes Biomacromolecules 2005 6 3357 3366 16283766 10.1021/bm0503726

[85] Dautzenberg H Polyelectrolyte complex formation in highly aggregating systems. 1. Effect of salt: polyelectrolyte complex formation in the presence of NaCl Macromolecules 1997 30 7810 7815

[86] Cousin F Gummel J Ung D Boué F Polyelectrolyte-protein complexes: structure and conformation of each specie revealed by SANS Langmuir 2005 21 9675 9688 16207052 10.1021/la0510174

[87] Wang X Li Y Wang Y-W Lal J Huang Q Microstructure of β-Lactoglobulin/pectin coacervates studied by small angle neutron scattering J. Phys. Chem. B 2007 111 515 520 17228908 10.1021/jp0632891

[88] Kiriy A Yu J Stamm M Interpolyelectrolyte complexes: a single-molecule insight Langmuir 2006 22 1800 1803 16460109 10.1021/la051908b

[89] Stanić V Arntz Y Richard D Affolter C Nguyen I Crucifix C Schultz P Baehr C Frisch B Ogier J Filamentous condensation of DNA induced by pegylated poly-L-lysine and transfection efficiency Biomacromolecules 2008 9 2048 2055 18572920 10.1021/bm800287z

[90] Leisner D Imae T Strutural evolution of an interpolyelectrolyte complex of charged dendrimers with poly-L-glutamic acid J. Phys. Chem. B 2004 108 1798 1804

[91] Menjoge AR Kayitmazer AB Dubin PL Jaeger W Vasenkov S Heterogeneity of polyelectrolyte diffusion in polyelectrolyte-protein coacervates: a ^1^H pulsed field gradient NMR study J. Phys.Chem. B 2008 112 4961 4966 18373375 10.1021/jp711515h

[92] Lindhoud S Norde W Cohen Stuart MA Reversibility and relaxation behavior of polyelectrolyte complex micelle formation J. Phys. Chem. B 2009 113 5431 5439 19334698 10.1021/jp809489f

[93] Izumrudov VA Zhiryakova MV Kudaibergenov SE Controllable stability of DNA containing polyelectrolyte complexes in water salt solutions Biopolymers 1999 52 94 108 10898855 10.1002/1097-0282(1999)52:2<94::AID-BIP3>3.0.CO;2-O

[94] Izumrudov VA Zhiryakova MV Stability of DNA containing interpolyelectrolyte complexes in water salt solutions Macromol. Chem. Phys 1999 200 2533 2540

[95] Zelikin A Trukhanova ES Putnam D Izumrudov VA Litmanovich AA Competitive reactions in solution of Poly-L-histidine, Calf thymus DNA, and synthetic polyanions: determining the binding constants of polyelectrolytes J. Am. Chem. Soc 2003 125 13693 13699 14599208 10.1021/ja036387y

[96] Křiž J Dautzenberg H Cooperative interactions of unlike macromolecules. 2: NMR and theoretical study of electrostatic binding of sodium poly(stryrenesulfonate)s to copolymers with variably distributed cationic groups J. Phys. Chem. A 2001 105 3846 3854

[97] Carlsson F Linse P Malmsten M Monte Carlo simulations of polyelectrolyte-protein complexation J. Phys. Chem. B 2001 105 9040 9049

[98] Manning GS Simple model for the binding of a polyelectrolyte to an oppositely charged surface J. Phys. Chem. B 2003 107 11485 11490

[99] da Silva FLB Lund M Jönsson B Åkesson T On the complexation of proteins and polyelectrolytes J. Phys. Chem. B 2006 110 4459 4464 16509749 10.1021/jp054880l

[100] Feng X Dubin PL Measurement of equilibrium binding of cationic micelles to apolyanion by membrane filtration Langmuir 2002 18 2032 2035

[101] Seyrek E Dubin PL Henriksen J Nonspecific electrostatic binding characteristics if the heparin-antithrombin interaction Biopolymers 2007 86 249 259 17385667 10.1002/bip.20731

[102] Bugalowski W Lohman T A general method of analysis of ligand-macromolecule equilibria using a spectroscopic signal from the ligand to monitor binding. Application to *Escherichia coli* single-strand binding protein-nucleic acid interactions Biochemistry 1987 26 3099 3106 3300771 10.1021/bi00385a023

[103] DeHaseth PL Gross CA Burgess RR Record MT Jr Measurement of binding constants for protein DNA interactions by DNA-cellulose chromatography Biochemistry 1977 16 4777 4783 911788 10.1021/bi00641a003

[104] Schwarz G Klose S Thermodynamic and kinetic studies on the cooperative binding of proflavine to linear polyanions Eur. J. Biochem 1972 29 249 256 5081616 10.1111/j.1432-1033.1972.tb01981.x

[105] Daune MP Interactions protéines-acides nucléiques. 1. Etude théorique de l’association Eur. J. Biochem 1972 26 207 211 5046043 10.1111/j.1432-1033.1972.tb01758.x

[106] Schwarz G Cooperative binding to linear biopolymers Eur. J. Biochem 1970 12 442 453 5440621 10.1111/j.1432-1033.1970.tb00871.x

[107] McGhee JD vonHippel PH Theoretical aspects of DNA-protein interactions: Co-operative and non-co-operative binding of large ligands to a one-dimensional homogeneous lattice J. Mol. Biol 1974 86 469 489 4416620 10.1016/0022-2836(74)90031-x

[108] Record TM Jr Lohman TM de Haseth PL Ion effects on ligand-nucleic acid interactions J. Mol. Biol 1976 107 145 158 1003464 10.1016/s0022-2836(76)80023-x

[109] Mascotti DP Lohman TM Thermodynamic extent of counterion release upon binding oligolysines to single-stranded nucleic acids Proc. Natl. Acad. Sci. USA 1990 87 3142 3146 2326273 10.1073/pnas.87.8.3142 PMC53850

[110] Mascotti DP Lohman TM Thermodynamics of single-stranded RNA binding to oligolysines containing tryptophan Biochemistry 1992 31 8932 8946 1382582 10.1021/bi00152a033

[111] Lohman TM Overman LB Ferrari ME Kozlov AG A highly salt dependant enthalpy change for *Escherichia coli* SSB protein-nucleic acid binding due to ion-protein interactions Biochemistry 1996 35 5272 5279 8611514 10.1021/bi9527606

[112] Mascotti DP Lohman TM Thermodynamics of oligoarginines binding to RNA and DNA Biochemistry 1997 36 7272 7279 9188729 10.1021/bi970272n

[113] Ball V Winterhalter M Schwinté P Lavalle Ph Voegel J-C Schaaf P Complexation mechanism of bovine serum albumin and poly(allylamine hydrochloride) J. Phys. Chem. B 2002 106 2357 2364

[114] Feng X Leduc M Pelton R Polyelectrolyte complex characterization with isothermal titration calorimetry and colloid titration Colloids Surf., A: Physicochem. Eng. Aspects 2008 317 535 542

[115] Bucur CB Sui Z Schlenoff JB Ideal mixing in polyelectrolyte complexes and multilayers: entropy driven assembly J. Am. Chem. Soc 2006 128 13690 13691 17044688 10.1021/ja064532c

[116] Klocek G Seelig J Melittin interaction with sulfated cell surface sugars Biochemistry 2008 47 2841 2849 18220363 10.1021/bi702258z

[117] Michaels AS Polyelectrolyte complexes Ind. Eng. Chem 1965 57 32 41

[118] Laugel N Betscha C Winterhalter M Voegel J-C Schaaf P Ball V Relationship between the growth regime of polyelectrolyte multilayers and the polyanion/polycation complexation enthalpy J. Phys. Chem. B 2006 110 19443 19449 17004803 10.1021/jp062264z

[119] Boyd GE Wilson DP Manning GS Enthalpies of mixing polyelectrolytes with simple aqueous electrolyte solutions J. Phys. Chem 1976 80 808 810

[120] Ross PD Shapiro JT Heat of interaction of DNA with polylysine, spermine and Mg^2+^ Biopolymers 1974 13 415 416 4820071 10.1002/bip.1974.360130218

[121] Matulis D Rouzina I Bloomfield VA Thermodynamics of DNA binding and condensation: isothermal titration calorimetry and electrostatic mechanism J. Mol. Biol 2000 296 1053 1063 10686103 10.1006/jmbi.1999.3470

[122] Spink CH Chaires JB Thermodynamics of the binding of a cationic lipid to DNA J. Am. Chem. Soc 1997 119 10920 10928

[123] Ball V Winterhalter M Perret F Esposito G Coleman AW p-Sulfonatocalix[6]arene is an effective coacervator of poly(allylamine htdrochloride) Chem. Comm 2001 7 2276 2277 12240148 10.1039/b106361h

[124] Memmi L Lazar A Brioude A Ball V Coleman AW Protein-calixarene interactions: complexation of Bovine serum albumin by sulfonatocalix[n]arenes Chem Comm 2001 2474 2475 12240021 10.1039/b109190p

[125] Heerklotz H The microcalorimetry of lipid membranes J. Phys. Condens. Matter 2004 16 R441 R467

[126] Seelig J Titration calorimetry of lipid-peptide interactions Biochim. Biphys. Acta 1997 1331 103 116 10.1016/s0304-4157(97)00002-6 9325437

[127] Seelig J Thermodynamics of lipid-peptide interactions Biochim. Biophys. Acta 2004 1666 40 50 15519307 10.1016/j.bbamem.2004.08.004

[128] Bäuerle H-D Seelig J Interaction of charged and uncharged calcium channel antagonists with phospholipid membranes. Binding equilibrium, binding enthalpy and membrane location Biochemistry 1991 30 7203 7211 1830218 10.1021/bi00243a023

[129] Seelig J Ganz P Nonclassical hydrophobic effect in membrane binding equilibria Biochemistry 1991 30 9354 9359 1832558 10.1021/bi00102a031

[130] Gerebtzoff G Li-Blatter X Fischer H Frentzel A Seelig A Halogenation of drugs enhances membrane binding and permeation ChemBioChem 2004 5 676 684 15122640 10.1002/cbic.200400017

[131] Anderson TG Tan A Ganz P Seelig J Calorimetric measurement of phospholipid interaction with methyl-β-cyclodextrin Biochemistry 2004 43 2251 2261 14979721 10.1021/bi0358869

[132] Meier M Li Blatter X Seelig A Seelig J Interactions of verapamil with lipid membranes and P-Glycoprotein: connecting thermodynamics and membrane structure with functional activity Biophys. J 2006 91 2943 2955 16877510 10.1529/biophysj.106.089581 PMC1578493

[133] Machaidze G Ziegler A Seelig J Specific binding of Ro 09-O198 (cynnamycin) to phosphatidylethanol amine:a thermodynamic analysis Biochemistry 2002 41 1965 1971 11827543 10.1021/bi015841c

[134] Wenk M Seelig J Magainin 2 amide interaction with lipid membranes: calorimetric detection of peptide binding and pore formation Biochemistry 1998 37 39909 3916 10.1021/bi972615n 9521712

[135] Wieprecht T Beyermann M Seelig J Thermodynamics of the coil-helix transition of amphipatic peptides in a membrane environment: the role of vesicle curvature Biophys. Chem 2002 96 191 201 12034440 10.1016/s0301-4622(02)00025-x

[136] Meier M Seelig J Length dependence of the coil ⇔ β-sheet transition in a membrane environement J. Am. Chem. Soc 2008 130 1017 1024 18163629 10.1021/ja077231r

[137] Meier M Seelig J Thermodynamics of the coil↔β sheet transition in a membrane environment J. Mol. Biol 2007 369 277 289 17412361 10.1016/j.jmb.2007.02.082

[138] Hammel M Schwarzenbacher R Gries A Kostner GM Lagner P Prassl R Mechanism of the interaction of β2-Glycoprotein I with negatively charged phospholipid membranes Biochemistry 2001 40 14173 14181 11714270 10.1021/bi0114372

[139] Gabriel GJ Pool JG Som A Dabkowski JM Coughlin EB Muthukumar M Tew GN Interactions between antimicrobial polynorbornenes and phospholipid vesicles monitored by light scattering and microcalorimetry Langmuir 2008 24 12489 12495 18841926 10.1021/la802232p

[140] Scott MJ Jones MN The interaction of phospholipid liposomes with zinc citrate particles: a microcalorimetric investigation Colloids Surf., A: Phys. Engineer. Aspects 2001 182 247 256

[141] Schwarz G Damian L Winterhalter M Model-free analysis of binding at lipid membranes employing micro-calorimetric measurements Eur. Biophys. J 2007 36 571 579 17345079 10.1007/s00249-007-0143-5

[142] McLaughlin S The electrostatic properties of membranes Ann. Rev. Biophys. Biophys. Chem 1989 18 113 116 2660821 10.1146/annurev.bb.18.060189.000553

[143] Kabanov VA Yaroslavov AA What happens to negatively charged lipid vesicles upon interacting with polycation species? J. Controlled Release 2002 78 267 271 10.1016/s0168-3659(01)00496-5 11772467

[144] Volodkin D Ball V Voegel J-C Möhwald H Complexation of phosphocholine liposomes with polylysine. Stabilization by surface coverage or aggregation? Biochim. Biophys. Acta, Biomembr 2007 1768 280 290 10.1016/j.bbamem.2006.09.015 17084808

[145] Nebel S Bartoldus I Stegmann T Calorimetric detection of influenza virus induced membrane fusion Biochemistry 1995 34 5705 5711 7727430 10.1021/bi00017a001

[146] Basiuk VA Thermodynamics of adsorption of amino acids, small peptides, and nucleic acid components on silica adsorbents Biopolymers at Interfaces Surfactant Science Series Malmsten M Marcel Dekker New York. NY, USA 2003 volume 113 45 70

[147] Norde W Lyklema J Why proteins prefer interfaces J. Biomater. Sci. Polym. Ed 1992 3 183 202 10.1080/09205063.1991.9756659 1854684

[148] Norde W Lyklema J The adsorption of human plasma albumin and bovine pancreas ribonuclease at negatively charged polystyrene surfaces: V. Microcalorimetry J. Colloid Interface Sci 1978 66 295 302

[149] Norde W Lyklema J Thermodynamics of protein adsorption. Theory with special reference to the adsorption of Human Plasma Albumin and Bovine pancreas ribonuclease at polystyrene surfaces J. Colloid Interface Sci 1979 71 350 366

[150] Brandes N Welzel PB Werner C Kroh LW Adsorption-induced conformational changes of proteins onto ceramic particles: differential scanning calorimetry and FTIR analysis J. Colloid Interface Sci 2006 299 56 69 16500671 10.1016/j.jcis.2006.01.065

[151] Larsericsdotter H Oscarsson S Buijs J Thermodynamic analysis of lysozyme adsorbed to silica J. Colloid Interface Sci 2004 276 261 268 15271551 10.1016/j.jcis.2004.03.056

[152] Lin F-Y Chen WY Sang L-C Microcalorimetric studies of the interactions of lysozyme with immobilized metal ions: effects of ion, pH value, and salt concentration J. Colloid Interface Sci 1999 214 373 379 10339377 10.1006/jcis.1999.6193

[153] Lin F-Y Chen W-Y Hearn MTW Microcalorimetric studies on the interaction mechanism between proteins and hydrophobic solid surfaces in hydrophobic interaction chromatography: effects of salt, hydrophobicity of the solvent, and the structure of the protein Anal. Chem 2001 73 3875 3883 11534710 10.1021/ac0102056

[154] Lin FY Chen WY Hearn MTW Thermodynamic analysis of the interaction between proteins and solid surfaces: application to liquid chromatography J. Mol. Recognit 2002 15 55 93 11954053 10.1002/jmr.564

[155] Lee VA Craig RG Filisko FE Zand R Microcalorimetry of the adsorption of lysozyme onto polymeric substrates J. Colloid Interface Sci 2005 288 6 13 15927555 10.1016/j.jcis.2005.02.057

[156] Chen W-Y Liu Z-C Lin P-H Fang C-I Yamamoto S The hydrophobic interactions of the ion-exchanger resin ligands with proteins at high salt concentrations by adsorption isotherms and isothermal titration calorimetry Sep. Purif. Technol 2007 54 212 219

[157] Goobes R Goobes G Campbell CT Stayton PS Thermodynamics of statherin adsorption onto hydroxyapatite Biochemistry 2006 45 5576 5586 16634639 10.1021/bi052321z

[158] De M You C-C Srivastava S Rotello VM Biomimetic interactions of proteins with functionalized nanoparticles: a thermodynamic study J. Am. Chem. Soc 2007 129 10747 10753 17672456 10.1021/ja071642q

[159] Xu C-P van de Belt-Gritter B Busscher HJ van der Mei HC Norde W Calorimetric comparison of the interaction between salivary proteins and *Streptococcus Mutans* with and without antigen I/II Colloids Surf., B: Biointerfaces 2007 54 193 199 17140773 10.1016/j.colsurfb.2006.10.016

[160] Xie C-L Tang HK Song ZH Qu S-S Microcalorimetric study of bacterial growth Thermochim. Acta 1988 123 33 41

[161] Zhao R Liu Y Xie Z Shen P Qu S A calorimetric method for studying the biological effects of La^3+^ on *Escherichia Coli* J. Biochem. Biophys. Methods 2000 46 1 9 11086189 10.1016/s0165-022x(00)00124-x

